# USP9X regulates the proliferation, survival, migration and invasion of gastric cancer cells by stabilizing MTH1

**DOI:** 10.1186/s12876-024-03321-9

**Published:** 2024-07-29

**Authors:** Wenji Xu, Yaping Zhang, Yingrui Su, Libin Li, Xinxia Yang, Lixing Wang, Hongzhi Gao

**Affiliations:** 1 Digestive System Department, The Second Affiliated Hospital of Fujian Medical University, Quanzhou, 362000 China; 2 Central Laboratory, The Second Affiliated Hospital of Fujian Medical University, No. 34, Zhongshan North Road, Licheng District, Quanzhou, 362000 China; 3 Nuclear Medicine Department, The Second Affiliated Hospital of Fujian Medical University, Quanzhou, 362000 China; 4 Neurosurgery Department, The Second Affiliated Hospital of Fujian Medical University, Quanzhou, 362000 China

**Keywords:** MTH1, USP9X, Deubiquitination, Proliferation

## Abstract

**Background:**

MutT homolog 1 (MTH1) sanitizes oxidized dNTP pools to promote the survival of cancer cells and its expression is frequently upregulated in cancers. Polyubiquitination stabilizes MTH1 to facilitate the proliferation of melanoma cells, suggesting the ubiquitin system controls the stability and function of MTH1. However, whether ubiquitination regulates MTH1 in gastric cancers has not been well defined. This study aims to investigate the interaction between MTH1 and a deubiquitinase, USP9X, in regulating the proliferation, survival, migration, and invasion of gastric cancer cells.

**Methods:**

The interaction between USP9X and MTH1 was evaluated by co-immunoprecipitation (co-IP) in HGC-27 gastric cancer cells. siRNAs were used to interfere with USP9X expression in gastric cancer cell lines HGC-27 and MKN-45. MTT assays were carried out to examine the proliferation, propidium iodide (PI) and 7-AAD staining assays were performed to assess the cell cycle, Annexin V/PI staining assays were conducted to examine the apoptosis, and transwell assays were used to determine the migration and invasion of control, USP9X-deficient, and USP9X-deficient plus MTH1-overexpressing HGC-27 and MKN-45 gastric cancer cells.

**Results:**

Co-IP data show that USP9X interacts with and deubiquitinates MTH1. Overexpression of USP9X elevates MTH1 protein level by downregulating its ubiquitination, while knockdown of USP9X has the opposite effect on MTH1. USP9X deficiency in HGC-27 and MKN-45 cells causes decreased proliferation, cell cycle arrest, extra apoptosis, and defective migration and invasion, which could be rescued by excessive MTH1.

**Conclusion:**

USP9X interacts with and stabilizes MTH1 to promote the proliferation, survival, migration and invasion of gastric cancer cells.

## Introduction

Gastric cancer (GC) is a highly heterogeneous disease and causes the third most cancer-related deaths worldwide [1–3]. It is well known that excessive reactive oxygen species (ROS) and DNA damage hinder GC development [3]. Increased levels of ROS induce DNA damage via oxidizing the mitochondrial deoxynucleoside triphosphate pool, thus reducing the survival of cancer cells [3]. Meanwhile, high ROS levels can also repress tumor development by inhibiting proliferation and inducing apoptosis of cancer cells [4–8]. ROS-induced apoptosis requires the incorporation of 8-oxodeoxy-guanine (8-oxo-dGTP) and 2-OH-deoxy-adenosine (2-OH-dATP) into genomic DNA, a process that is negatively regulated by mutT homolog 1(MTH1) [4, 9–12].

The expression of MTH1, also known as NUDT1, is upregulated in multiple types of digestive tract tumors [3, 13], and high MTH1 expression promotes the survival of tumor cells but not normal cells [4, 14], making MTH1 a potential target for cancer treatment [15–17]. However, the molecular mechanisms that regulate MTH1 expression are not well understood. Recent studies report that the K63-linked polyubiquitination of MTH1 by the corresponding ubiquitin ligase complex stabilizes MTH1 in melanoma cells [18], suggesting that the ubiquitin system, which is frequently dysregulated in various cancers [19, 20], may control the expression and function of MTH1.

USP9X is a deubiquitinase that regulates a variety of cellular processes such as cell proliferation, migration, apoptosis, and autophagy [21, 22]. USP9X is involved in the tumorigenesis of various types of cancers [22–25], and high expression of USP9X is found and associated with poor prognosis of GC [26]. USP9X modulates K63-linked ubiquitination of its target proteins in multiple contexts [27, 28], but whether it controls GC development and affects MTH1 stability remains elusive.

In this study, we investigated the interaction between USP9X and MTH1 in HEK293T cells and HGC-27 GC cells. We then explored the role of USP9X in proliferation, cell cycle, apoptosis, migration, and invasion of HGC-27 and MKN-45 GC cells. We also assessed whether the effects of USP9X deficiency in these cells could be rescued by MTH1 overexpression.

## Materials and methods

### Plasmid constructions

cDNAs encoding wildtype and mutant USP9X, and MTH1, were amplified via high fidelity polymerase chain reaction (PCR) and subcloned into pCMV-HA and pCMV-Flag (Clontech, USA), and were named HA-USP9X, HA-USP9X C1566A, and Flag-MTH1, respectively. The primers used for plasmid construction are listed in Table 1. Myc-ubiquitin (Myc-Ub) plasmid was purchased from XIAMEN Anti-HeLa Biological Technology Trade Co., Ltd, China. The transfection was carried out by Lipofectamine 2000 (Invitrogen, USA) according to the manufacturer’s guide.

**Table 1:** Primers for plasmid constructions

Name	Sequence (5′-3′)
HA-USP9X-F	CTAGAGAATTCGGATCCATGACAGCCACGACTCGTGGCTC
HA-USP9X-R	AGCTTCCATGGCTCGAGTTGATCCTTGGTTTGAGGTGG
HA-USP9X C1566A-F	CCGGTGCTACTGCTTACATGAATTCTGTGATTC
HA-USP9X C1566A-R	CATGTAAGCAGTAGCACCGGCATTTTTCAG
Flag-MTH1-F	CTAGAGAATTCGGATCCATGGGCGCCTCCAGGCTC
Flag-MTH1-R	GCTTCCATGGCTCGAGGACCGTGTCCACCTCGCGGAGTG

F: forward primer; R: reverse primer

### Cell culture

HEK293T, HGC-27, and MKN-45 cells were obtained from IMMOCELL (Xiamen, China) and cultured with Dulbecco’s minimal essential medium (Gibco, USA) supplemented with 10% fetal bovine serum (HyClone, USA), 100 IU penicillin, and 100 µg/mL streptomycin in an incubator with 5% CO_2_ at 37 °C.

### RNA interference

To knock down USP9X expression, we designed three small interfering RNAs (siRNAs) including siUSP9X-1: 5′-AGAAATCGCTGGTATAAAT-3′; siUSP9X-2: 5′-ACACGATGCTTTAGAATTT-3′ and siUSP9X-3: 5′-GTACGACGATGTATTCTCA-3′, which were synthesized by Huzhou Hippo Biotechnology Co., Ltd, China. The transfection was carried out by Lipofectamine 2000 following the manufacturer’s instructions.

### Western blot

Cells were collected in lysis buffer containing 50 mM Tris-HCl (pH 7.4), 150 mM NaCl, 5 mM MgCl_2_, 1 mM ethylene diamine tetraacetic acid, 1% Triton X-100, 10% glycerol, and 1 mM phenylmethylsulfonyl fluoride (Beyotime, China). A BCA kit (Beyotime) was used for protein quantification. Protein extracts or immunoprecipitated samples were separated using 10–12% sodium dodecyl sulfate-polyacrylamide gel electrophoresis (SDS-PAGE), transferred onto PVDF membranes (Millipore, USA), and trimmed along the lane boundaries. After blocking with 5% skim milk for 1 h at 25 °C, the membranes were probed with antibodies against HA tag (catalog number: 51064-2-AP, Proteintech, China), Flag tag (catalog number: 20543-1-AP, Proteintech), Myc tag (catalog number: 16286-1-AP, Proteintech), beta-actin (catalog number: 20536-1-AP, Proteintech), MTH1 (catalog number: 16705-1-AP, Proteintech), USP9X (catalog number: 55054-1-AP, Proteintech), or ubiquitin (catalog number: 10201-2-AP, Proteintech) at 4 ℃ overnight, followed by incubation with horseradish peroxidase-conjugated goat anti-rabbit IgG (catalog number: SA00001-2, Proteintech) for 1 h at room temperature. Subsequently, the signals were visualized using ECL chemiluminescence (Thermo Fisher Scientific, USA).

### Co-immunoprecipitation (co-IP) assay

The protein samples were incubated with antibodies against Flag tag (catalog number: 66008-3-Ig, Proteintech) or MTH1 (catalog number: sc-373,709, Santa Cruz Biotechnology, USA), overnight at 4 °C. Then the samples were incubated with protein A + G agarose beads (catalog number: P2055-10 ml, Beyotime) for 4 h at 4 °C. After washing with lysis buffer five times, the precipitated beads were boiled for 10 min in SDS-PAGE protein loading buffer (catalog number: P0015A, Beyotime), and the supernatant was collected for Western blot analysis.

### Real-time qPCR (RT qPCR)

An RNA Simple Total RNA Kit (catalog number: DP419, Tiangen Biotech, China) was used to extract total RNA from cells following the manufacturer’s instructions. First-strand cDNA Synthesis kit (Toyobo, Japan) was used to reverse transcribe RNA to cDNA. RT qPCR was conducted with Power SYBR Green PCR Master Mix (Applied Biosystems, USA) on ABI 7500 Real-Time PCR System (Applied Biosystems). The primers used were as follows: MTH1 forward primer, 5′-GTCTTCTGCACAGACAGCATCC-3′; MTH1 reverse primer, 5′-CTGAAGCAGGAGTGGAAACCAG-3′; GAPDH forward primer: 5′-GACATCAAGAAGGTGGTGAA-3′ and GAPDH reverse primer: 5′-TGTCATACCAGGAAATGAGC-3′.

### Ubiquitination assay

Flag-MTH1 and Myc-Ub were co-transfected with HA-USP9X or HA-USP9X C1566A into HGC-27 cells, and siUSP9X was transfected into HGC-27 cells. After 36 h, the cells were treated with 5 µM MG132 for 3 h before co-IP and Western blot analysis.

### MTT assay

HGC-27 or MKN-45 cells were seeded into 96-well plates (1 × 10^5^ cells per well) and were co-transfected with siUSP9X and Flag-MTH1 plasmids. After 48 h, the cells were rinsed and tested using the MTT assay kit (catalog number: C0009S, Beyotime) according to the manufacturer’s instructions, at indicated time points.

### Cell cycle assay

HGC-27 or MKN-45 cells transfected with siUSP9X and Flag-MTH1 plasmids were cultured for 48 h in 6-well plates. Then the cells were sequentially treated with 70% alcohol, 0.1% Triton X-100/PBS with 10 µg/mL RNase, and 50 µg/mL propidium iodide (catalog number: ST511, Beyotime) at 4 °C for 4 h, 37 °C for 30 min, and 25 °C for 30 min, respectively. A flow cytometer was used to test the treated cells.

### 7-amino-actinomycin D (7-AAD) assay

The cells were suspended, pelleted, and fixed in pre-chilled 95% ethanol at -20 °C for 12 h. Subsequently, they were washed with PBS and incubated with the 20 mg/mL 7-AAD (Beyotime) in PBS for 10 min at 37 °C. Finally, the cells were analyzed with flow cytometry and the FlowJo software (v10.8.1) was used to analyze the data.

### Annexin V/PI-FITC staining

Apoptosis of GC cells was examined by the Annexin V-FITC kit (Beyotime) according to the manufacturer’s guide. Briefly, the cells were resuspended and pelleted by centrifugation at 200 g for 5 min and washed twice with PBS. Annexin V-FITC/PI staining solution and binding buffer were added to the cells and mixed gently. After that, the cells were incubated in the dark for 20 min at 25 °C. Then the cells were measured using flow cytometry.

### Transwell assay

HGC-27 or MKN-45 cells transfected with siUSP9X and Flag-MTH1 plasmids were cultured for 48 h, and cell invasion and migration were measured using 24-well Transwell plates with and without Matrigel separately. Cells (1 × 10^5^ cells per well) in 100 µL DMEM without FBS were seeded into the upper chamber. DMEM (500 µL) containing 10% FBS was added to the lower chamber. After 24 h, 0.5% crystal violet was used to stain the invasive cells for 30 min at 25 °C and the results were photographed using a light microscope (Motic, China).

### Statistical analysis

The data were analyzed using SPSS software (v22.0) and presented as the mean ± standard deviation. The Mann-Whitney test was used for non-parametric data between the two groups. Unpaired two-tailed Student’s t-test was used to determine the significance of parametric data between the two groups. One-way ANOVA followed by Tukey’s post-hoc test was performed to determine the significance of differences among multiple groups. 

## Results

### USP9X interacts with MTH1 in HEK293T and HGC-27 GC cells

**Fig. 1:**
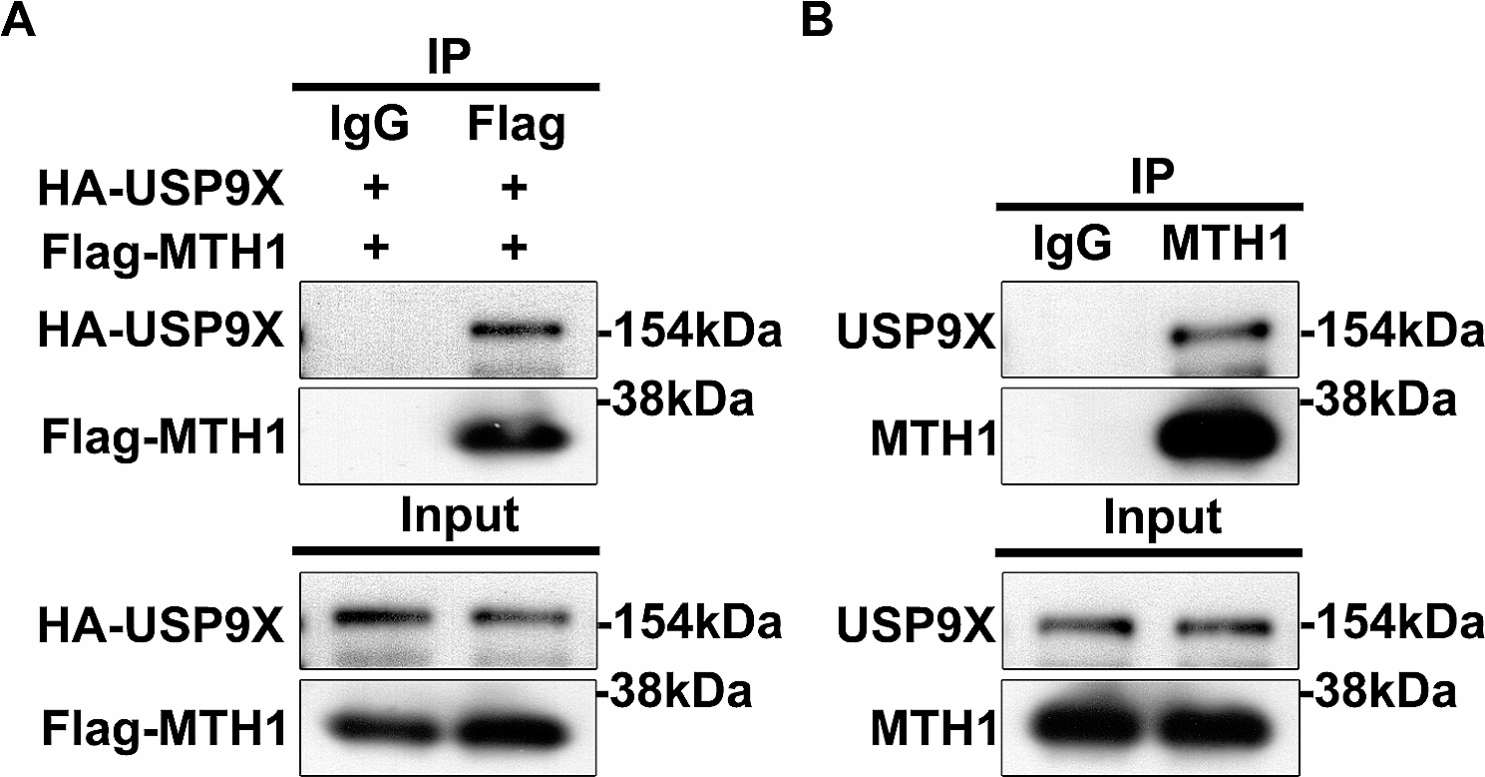
USP9X interacts with MTH1. (**A**) Co-IP assay data show that exogenous USP9X and MTH1 interact with each other in HEK293T cells. (**B**) Co-IP assay shows that endogenous USP9X interacts with MTH1 in HGC-27 cells

To determine whether USP9X interacts with MTH1, we overexpressed USP9X and MTH1 in HEK293T cells and performed co-IP assays. We found that exogenous USP9X and MTH1 directly interact with each other in HEK293T cells (Fig. 1A). To find out whether such an interaction exists in GC cells, we performed co-IP using HGC-27 cells that express endogenous USP9X and MTH1 expression, and we also detected the interactions between the two proteins (Fig. 1B). These results demonstrate that USP9X interacts with MTH1, and this interaction may exist among different cell types.

### USP9X stabilizes MTH1 in HGC-27 cells

**Fig. 2:**
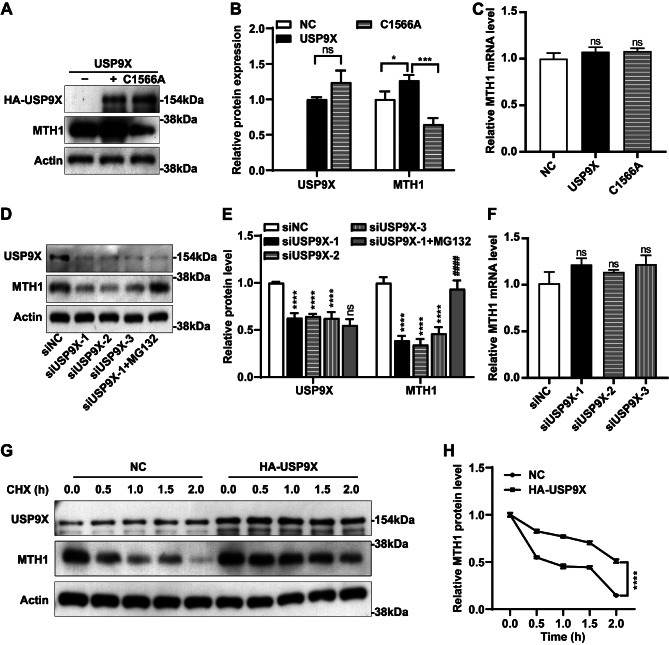
USP9X regulates MTH1 stability. (**A**) Western blot data show that overexpression of wildtype, but not mutant USP9X increases MTH1 protein level in HGC-27 cells. (**B**) Quantification of the Western blot data displayed in (**A**). *P < 0.05, ***P < 0.001, ns: not significant. (**C**) The mRNA levels of MTH1 are not altered by USP9X overexpression. ns: not significant. (**D**) Western blot data reveal that USP9X knockdown significantly reduces MTH1 protein levels, but such inhibition can be reversed by treatment of MG132. (**E**) Quantification of the western blot results displayed in (**D**). ****P < 0.0001 vs siNC; ####*P* < 0.0001, ns: not significant vs siUSP9X-1. (**F**) RT qPCR results indicate that the mRNA levels of MTH1 are not changed by USP9X knockdown. ns: not significant. (**G**) Western blot results show that USP9X overexpression enhances the stability of MTH1 in HGC-27 cells. (**H**) Quantification of the western blot data displayed in (**G**). ****P < 0.0001. NC: negative control

To explore the potential regulatory effect of USP9X on MTH1, we overexpressed wildtype USP9X or USP9X mutant (USP9X C1566A), which lacks the catalytic function of USP, in HGC-27 cells. We observed that overexpression of wildtype but not mutant USP9X results in an increased level of MTH1 compared to the negative control (Fig. 2A and B), and this regulatory effect is post-translational as the mRNA levels of MTH1 remains unchanged by excessive USP9X (Fig. 2C). We then depleted USP9X in HGC-27 cells by siRNAs and found that MTH1 protein level was significantly decreased upon USP9X knockdown, and this inhibition could be reversed by treatment of a proteasome inhibitor MG-132 (Fig. 2D and E), which blocks ubiquitination-mediated protein degradation [29]. Consistently, the mRNA level of MTH1 was not altered by USP9X deficiency (Fig. 2F). To decide whether USP9X regulates MTH1 stability, we treated HGC-27 cells overexpressing USP9X with a protein synthesis inhibitor, cycloheximide (CHX), and examined the levels of MTH1 protein at different time points. We found that excessive USP9X significantly enhances the stability of MTH1 (Fig. 2G and H). Taken together, these observations indicate that USP9X controls the stability of MTH1 in HGC-27 cells.

### USP9X deubiquitinates MTH1 in HGC-27 cells

**Fig. 3:**
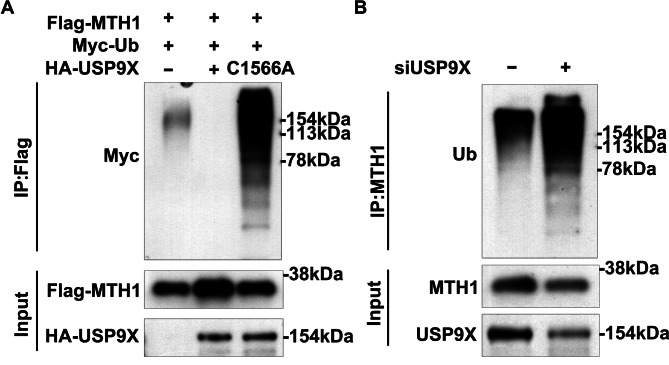
USP9X deubiquitinates MTH1 in HGC-27 cells. (**A**) Western blot data show that overexpression of wildtype USP9X reduces the ubiquitination of MTH1, while overexpression of mutant USP9X significantly elevates the ubiquitination of MTH1. (**B**) Western blot data reveal that USP9X knockdown results in an increased level of MTH1 ubiquitination. Ub: ubiquitin

Since USP9X is a deubiquitinase, we then asked whether USP9X directly controls the ubiquitination of MTH1. We introduced Myc-Ub and purified ubiquitinated MTH-1 from control and USP9X-expressing HGC-27 cells. We observed that overexpression of wildtype USP9X significantly reduced the ubiquitination of MTH1, while mutant USP9X had the opposite function (Fig. 3A). Conversely, knockdown of USP9X greatly upregulated MTH1 ubiquitination (Fig. 3B). These data reveal that USP9X directly deubiquitinates MTH1 in HGC-27 cells.

### USP9X deficiency causes reduced proliferation, cell cycle arrest, and increased apoptosis in HGC-27 and MKN-45 cells, which can be rescued by MTH1 overexpression

**Fig. 4:**
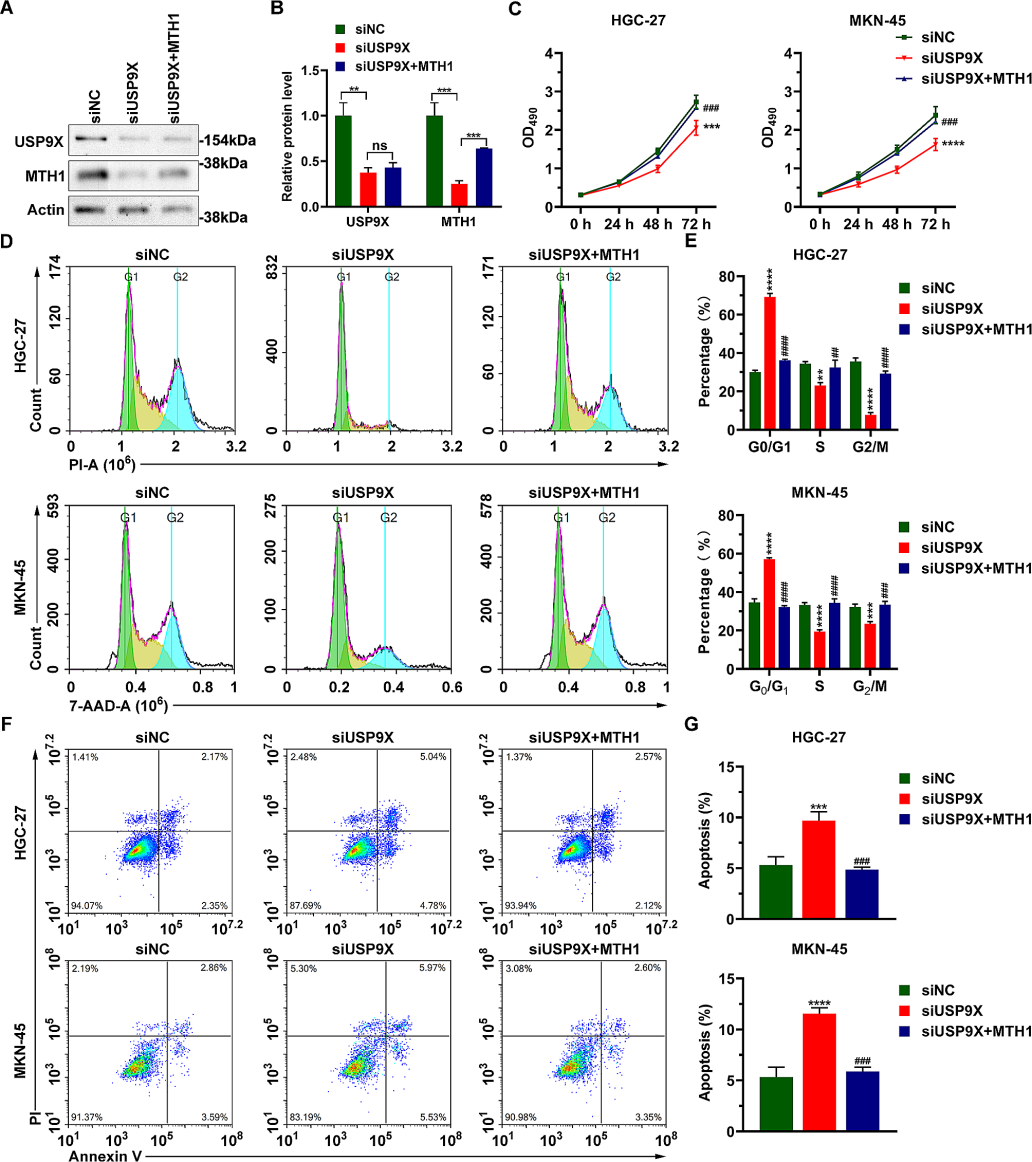
USP9X controls the proliferation and survival of HGC-27 and MKN-45 cells. (**A**) Western blot data show that USP9X and MTH1 expression is reduced by USP9X knockdown. (**B**) Quantification of the western blot data displayed in (**A**). **P < 0.01, ***P < 0.001, ns: not significant. (**C**) MTT assay results show that USP9X knockdown downregulates the proliferation of HGC-27 and MKN-45, which can be rescued by MTH1 overexpression. (**D**) PI and 7-AAD staining data reveal that USP9X deficiency causes cell cycle arrest in GC cells, but this could be reversed by excessive MTH1. (**E**) Quantification of the data displayed in (**D**). (**F**) Annexin V/PI staining results show that apoptosis is increased by USP9X knockdown but is restored to a normal level in the presence of excessive MTH1. (**G**) Quantification of the data displayed in (**F**). **P < 0.01, ***P < 0.001, ****P < 0.0001 vs siNC; ##*P* < 0.01, ###*P* < 0.001, ####*P* < 0.0001 vs siUSP9X. NC: negative control

To investigate whether USP9X controls the proliferation of GC cells, we reduced USP9X expression in HGC-27 and MKN-45 cells with siRNAs and found both USP9X and MTH1 protein levels are downregulated by USP9X knockdown (Fig. 4A and B). To assess the effect of USP9X on GC cell proliferation, we carried out MTT assays and observed that the proliferation is significantly reduced in USP9X-deficient GC cells compared with that of control cells (Fig. 4C). Similarly, the cell cycle was arrested in the G1 stage by USP9X knockdown (Fig. 4D and E), and extra apoptosis was identified in USP9X-deficient GC cells (Fig. 4F and G). Interestingly, all these defects caused by USP9X knockdown can be rescued by overexpression of MTH1 (Fig. 4C-G). Collectively, these findings indicate that USP9X controls the proliferation and survival of GC cells by stabilizing MTH1.

### USP9X regulates the migration and invasion of HGC-27 and MKN-45 cells by stabilizing MTH1

**Fig. 5:**
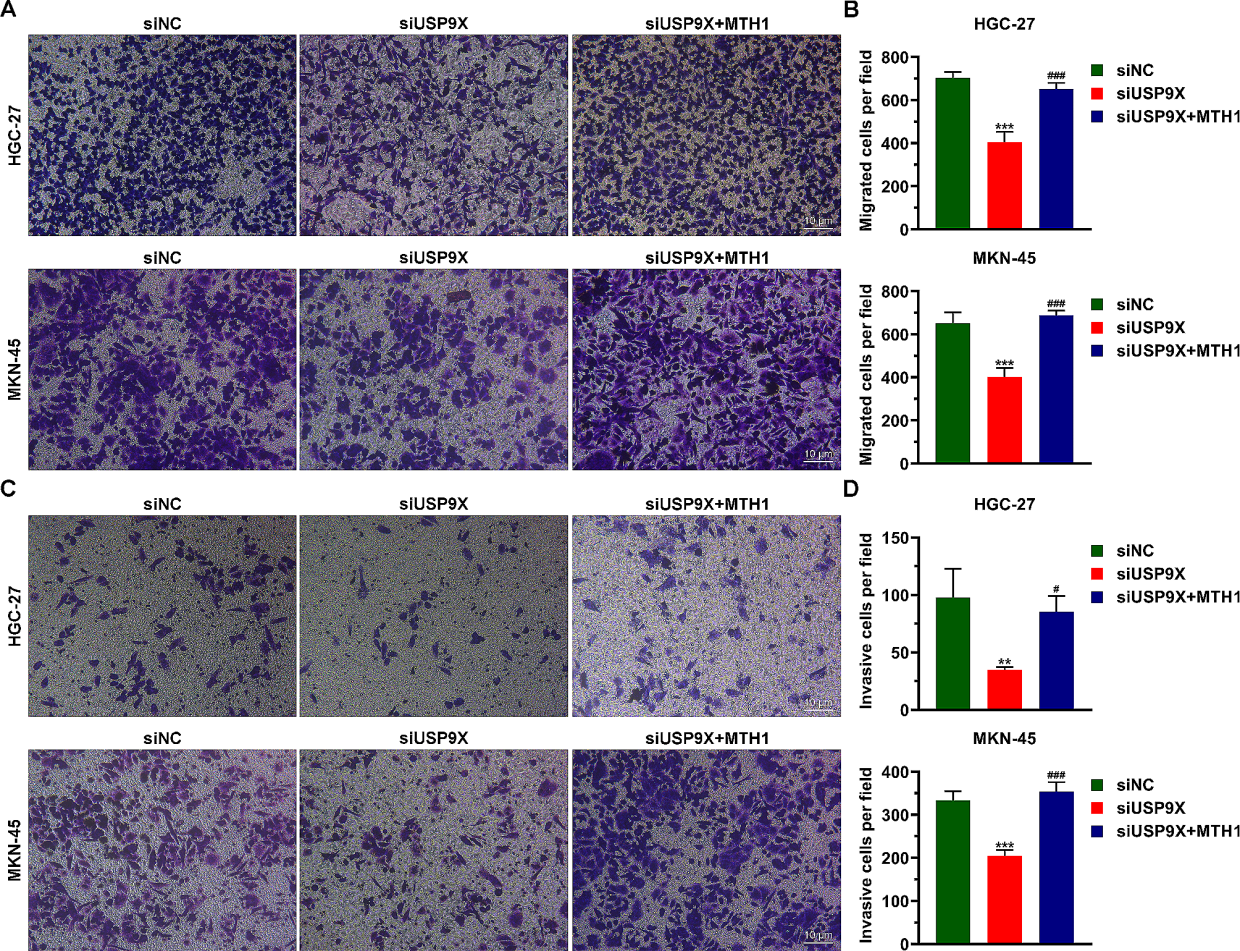
USP9X promotes the migration and invasion of HGC-27 and MKN-45 cells. (**A**) Transwell assay data show that the migration of GC cells was impaired by USP9X knockdown, and overexpression of MTH1 rescues the effect of USP9X deficiency on GC cell migration. (**B**) Quantification of the data in (**A**). (**C**) Transwell assay data show that the invasion of GC cells was mitigated by USP9X knockdown, and overexpression of MTH1 restores the invasion of USP9X-deficient GC cells. (**D**) Quantification of the data in (**C**). **P < 0.01, ***P < 0.001vs siNC; #*P* < 0.05, ###*P* < 0.001 vs siUSP9X. NC: negative control

To explore whether USP9X regulates GC cell migration and invasion, we performed transwell assays using control and USP9X-deficient HGC-27 and MKN-45 cells. We found that USP9X deficiency resulted in defective migration and invasion of these cells, and these defects could be efficiently reversed by excessive MTH1 (Fig. 5A-D). These findings demonstrate that USP9X regulates the migration and invasion of GC cells by stabilizing MTH1.

## Discussion

GC is a common gastrointestinal malignancy with a high recurrence rate and poor prognosis [2]. Various risk factors of GC, including unhealthy diet and *Helicobacter pylori* infection, can induce excessive ROS production, leading to 8-oxo-dGTP accumulation, DNA damage, carcinogenesis, and the transcription or post-translational modification of base excision and other DNA repair enzymes in GC [3]. Therefore, targeting DNA repair enzymes such as MTH1 may be an optional strategy for GC treatment. On the other hand, ROS-triggered oxidative stress and DNA damage promote cell death and senescence to repress cancer development [4, 5, 30]. Therefore, cancer cells protect themselves from ROS by enhancing redox reactions or activating oxidative damage repair pathways that are less active in normal cells [4, 31, 32].

MTH1 is an enzyme that sanitizes 8-oxo-dGTP accumulation through pyrophosphatase activity [18]. Overexpression of MTH1 is identified in various types of cancers including GC [3, 33]. However, the mechanisms by which cancer cells elevate MTH1 expression are unclear. Our finding that USP9X controls the stability of MTH1 expression suggests that post-translational modification of MTH1 proteins may be a key regulator of MTH1 expression and function. Nevertheless, whether USP9X removes MTH1’s K63-linked or other types of polyubiquitination needs further characterization. On the other hand, MTH1 is ubiquitinated by an E3 ligase SKP2 in melanoma cells, which plays an oncogenic role [18]. Therefore, it is intriguing to determine whether SKP2 or other E3 ligases that ubiquitinate MTH1 in GC cells contribute to GC development and progression.

Deubiquitinases (DUBs) can stabilize proteins by removing the ubiquitin chain on the substrate protein [25]. Among about 90 DUBs, USP9X plays diverse roles in different cancers by regulating the stability of its targets. For example, USP9X inhibits the formation of colon tumors by stabilizing FBW7 protein [25]. USP9X also promotes the progression of hepatocellular carcinoma by targeting β-catenin [34]. In addition, upregulation of USP9X can lead to the occurrence of breast cancer [35], and facilitates the progression of melanoma [36], esophageal squamous cell carcinoma [37, 38], and lung cancer [39, 40]. Although USP9X protein expression is upregulated in GC tissues and predicts poor prognosis [26], its biological function in GC is incompletely defined. Our observations that USP9X interacts with and deubiquitinates MTH1 in HGC-27 cells and controls the proliferation, survival, migration, and invasion of GC cells expand our knowledge about the function and target of USP9X in cancer biology. It is also interesting to study whether other proteins are controlled by USP9X in GC cells in the future.

MTH1 inhibitors have been developed as potential anti-cancer drugs to treat various cancers [4, 41, 42]. In GC, MTH1 inhibitors also exhibit anti-cancer effects [3, 43]. However, unwanted dose-dependent adverse effects of such inhibitors may limit their clinical application [42]. Moreover, inhibitors blocking the enzymatic activity of MTH1 have insufficient efficacy to kill cancer cells [44]. Consequently, targeting MTH1 stabilizers such as USP9X with inhibitors like WP1130 [45], may improve the efficacy of MTH1 inhibitors in combating GC. It is therefore attractive to test the effect of the combined application of MTH1 and USP9X inhibitors on GC cells.

Despite our in vitro experimental data uncovering a solid interaction between USP9X and MTH1 in GC cells, the role of USP9X in GC and their interaction in GC tissues need to be verified by in vivo experiments.

## Conclusion

In summary, our study reveals that USP9X can control the proliferation, survival, migration and invasion of GC cells by interacting with and deubiquitinating MTH1. Targeting MTH1 or USP9X may be an alternative option to treat GC patients.

## Data Availability

The data that support the findings of this study have been included in the manuscript and are available from the corresponding author upon reasonable request.

## Abbreviations

Co-IP: Co-immunoprecipitation
GC: Gastric cancer
ROS: Reactive oxygen species
MTT: 3-(4,5-dimethylthiazol-2-yl)-2,5-diphenyl-2 H-tetrazolium bromide
DUBs: Deubiquitinases
